# Efficient deformation mechanisms enable invasive cancer cells to migrate faster in 3D collagen networks

**DOI:** 10.1038/s41598-022-11581-2

**Published:** 2022-05-12

**Authors:** Laure Laforgue, Arnold Fertin, Yves Usson, Claude Verdier, Valérie M. Laurent

**Affiliations:** 1 Univ. Grenoble Alpes, CNRS, LIPhy, 38000 Grenoble, France; 2 Institute for Advanced Biosciences, INSERM U1209, CNRS UMR 5309, Univ. Grenoble Alpes, Grenoble, 38000 France; 3 Univ. Grenoble Alpes, CNRS, UMR 5525, VetAgro Sup, Grenoble INP, TIMC, 38000 Grenoble, France

**Keywords:** Biophysics, Cancer

## Abstract

Cancer cell migration is a widely studied topic but has been very often limited to two dimensional motion on various substrates. Indeed, less is known about cancer cell migration in 3D fibrous-extracellular matrix (ECM) including variations of the microenvironment. Here we used 3D time lapse imaging on a confocal microscope and a phase correlation method to follow fiber deformations, as well as cell morphology and live actin distribution during the migration of cancer cells. Different collagen concentrations together with three bladder cancer cell lines were used to investigate the role of the metastatic potential on 3D cell migration characteristics. We found that grade-3 cells (T24 and J82) are characterized by a great diversity of shapes in comparison with grade-2 cells (RT112). Moreover, grade-3 cells with the highest metastatic potential (J82) showed the highest values of migration speeds and diffusivities at low collagen concentration and the greatest sensitivity to collagen concentration. Our results also suggested that the small shape fluctuations of J82 cells are the signature of larger migration velocities. Moreover, the displacement fields generated by J82 cells showed significantly higher fiber displacements as compared to T24 and RT112 cells, regardless of collagen concentration. The analysis of cell movements enhanced the fact that bladder cancer cells were able to exhibit different phenotypes (mesenchymal, amoeboid). Furthermore, the analysis of spatio-temporal migration mechanisms showed that cancer cells are able to push or pull on collagen fibers, therefore producing efficient local collagen deformations in the vicinity of cells. Our results also revealed that dense actin regions are correlated with the largest displacement fields, and this correlation is enhanced for the most invasive J82 cancer cells. Therefore this work opens up new routes to understand cancer cell migration in soft biological networks.

## Introduction

Cell migration is fundamental for many biological processes such as the immune response, wound repair, tissue homeostasis and also for pathologies such as cancer metastasis, inflammation or autoimmunity. The migration mechanisms have been widely studied but were initially limited to 2D motion on various substrates simplifying cell-substrate interactions^1–3^. Studies over the last decades have allowed to identify the different factors regulating 2D cell movement: substrate rigidity^4,5^, substrate anisotropy^6^ or the effect of extracellular protein density^7^. These effects can be measured thanks to the substrate deformation (usually functionnalized gels) and subsequent forces obtained by Traction Force Microscopy (TFM)^8–10^. Following these works, two main migration modes have been identified in the literature, the mesenchymal and amoeboid types of motion^11^.

In 3D networks, efforts have been made thanks to the use of confocal microscopy, in particular fluorescent reflection techniques^12,13^ allowed to vizualise biological fibers—such as collagen—mimicking the physiological environment of the cells. Other studies showed that the two modes of migration (mesenchymal or amoeboid) observed on 2D substrates are also found during migration in 3D matrices^14–16^. The proteolysis-independent amoeboid mode generally describes a way used by migrating cells constantly developping protrusions and retracting them by changing their shape, in order to move forward. Interestingly, cells do not necessary use adhesion molecules such as integrins to migrate in 3D^17^. Regarding the mesenchymal mode, cells usually exhibit a highly polarized shape with the development of protrusions, the formation and pulling on adhesions, cell contraction, release of bonds at the rear and recycling of proteins^11^. In this mode, the molecular mechanisms involved are based on actin polymerization and acto-myosin contractility, this being modulated by the level of attachment^18^, leading to traction force generation. In contrast to cells migrating on 2D substrates, large F-actin protrusions like lamellipodia are less present in 3D situations^11,16,19^. Moreover, cells cultured in 3D matrices show sparse stress fiber structures, more complex F-actin organisation at the front of the cell^20^ and the acto-myosin cytoskeleton is mainly localised in the sub-membranous actin cortex. Interestingly, some in vivo studies revealed the role of cytoskeleton regulators and adhesion receptors in pathological cell motility. Alterations in regulation pathways of acto-myosin contraction or actin dynamics contribute to the ability of tumor cells to switch between a variety of strategies^20,21^. Thereby, migrating tumor cells can adopt diverse morphologies and actin organization depending on cancer type^22^. When adopting preferentially a migration mode or switching between different modes, cancer cells can adapt to their surrounding ECM, interact and modify the microenvironment for optimum motility^23^. Moreover, the temporal fluctuation of cancer cell shapes can facilitate their migration^24^. Taken together, these results highlight the complexity of 3D migration and the need to bring new insights about the spatio-temporal cell behavior^25^, in particular cell-induced 3D environment deformations.

This is why recent studies focused on the ability of cells to deform and remodel 3D matrices using natural biomaterials^26^. To measure traction forces exerted by cells at a given time, cells were first encapsulated in PEG containing fluorescent microbeads^27^. In collagen gels containing fluorescent beads, the strain energy exerted by invasive or non invasive cells on their surroundings showed complex and anisotropic patterns^28^. Using confocal reflection microscopy, local displacement fields^29^ measured in collagen networks surrounding breast carcinoma cells showed a surprising constant cell contractility regardless of the collagen matrix stiffness. Time-lapse imaging of migrating cells was also used to elucidate the molecular mechanisms underlying force transmission in 3D fibrin matrices^30^. Additionally, a few in vitro studies investigated the correlation between metastatic potential and migration of cancer cells in synthetic or physiological matrices. It was found that aggressive tumorigenic cells in 3D synthetic environments migrate differently as compared to normal primary cells^31^. Finally, breast cancer biomarkers in association with morphological characteristics appear to be the dominant factor influencing cell motility in compliant collagen matrices^26^.

Here, we addressed the role of cancer cell invasiveness on 3D motility, using three bladder cancer cell lines with different metastatic potentials^32^ in various collagen matrices, together with a recently developped method for calculating collagen displacements^33^. We determined migration speeds at the lowest collagen concentration and found a significant difference, as well as a collagen concentration-dependent persistence. Then, using cell morphology and sphericity index, we showed that grade-3 cells were able to migrate using long protrusions and elongated morphology whereas grade-2 cells move with a round or slightly elongated shape showing short extensions. Then we used the Phase Correlation method^33^ to calculate the collagen displacement fields resulting from forces exerted by cells, together with the detailed analysis of actin. This analysis showed that collagen remodelling was closely connected to cell shape changes, associated with several actin-rich regions, playing a fundamental role in 3D migration. Furthermore, our results emphasized the various mechanisms (mesenchymal and amoeboid) used by cancer cells to migrate through these soft gels.

## Results

### Effects of metastatic potential and collagen concentration on cell motility

**Figure 1:**
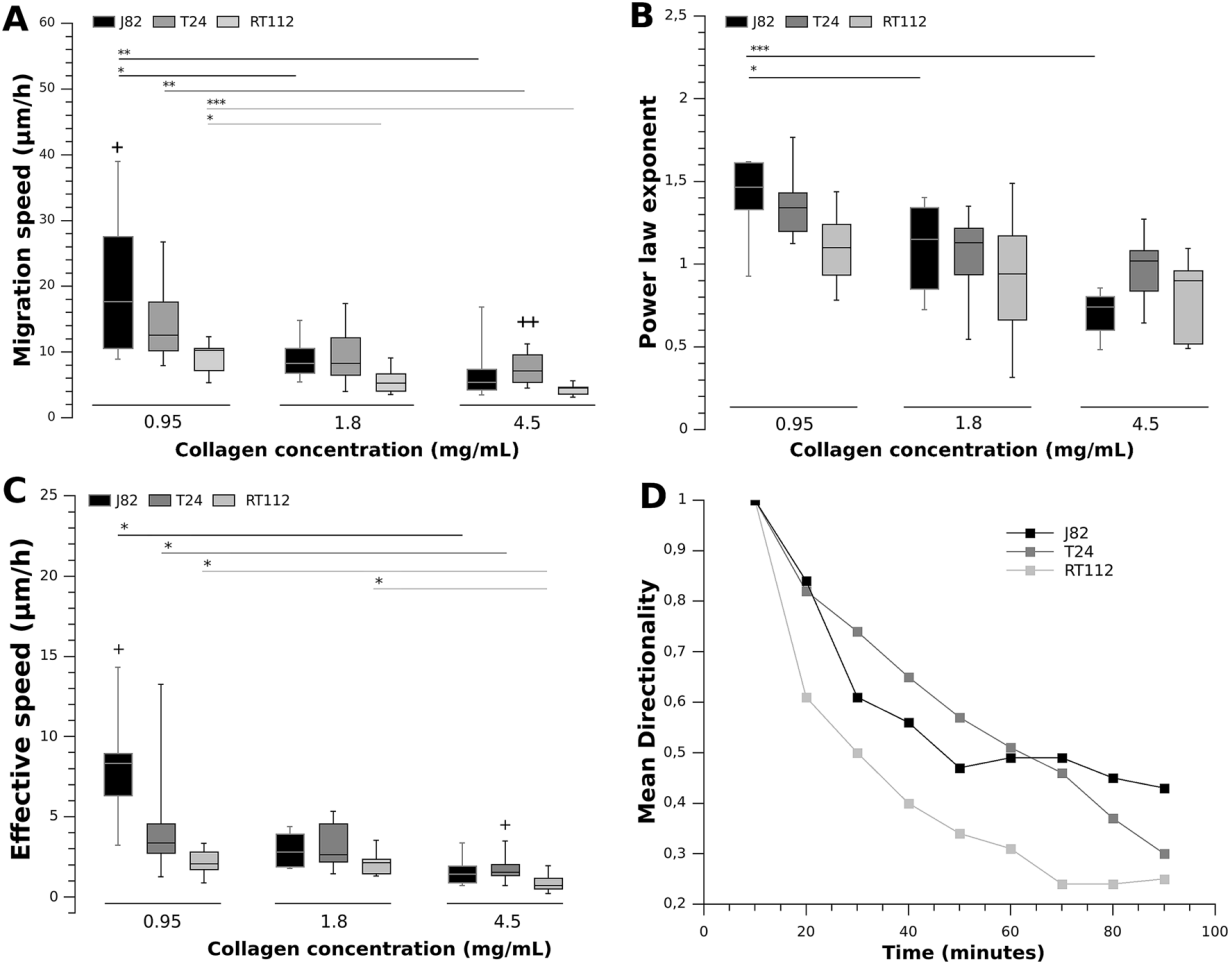
Effect of cell metastatic potential and collagen concentration on (**A**) migration speed, (**B**) power law exponent, (**C**) cell effective speed, (**D**) Mean directionality (at 0.95 mg/mL). Three cancer cell lines of increasing invasiveness (RT112 < T24 < J82) and collagen gel concentration (0.95 mg/mL, 1.8 mg/mL and 4.5 mg/mL) were used. Power-law exponents $\alpha$ in (**B**) were obtained from MSD(t) = D $(t/t_0)^\alpha$. Box-whisker plots indicate median value, 25% and 75% quartiles and whiskers extend to the 5% and 95% percentiles (**A**–**C**). Statistical significance from another collagen concentration * corresponds to p < 0.05, ** corresponds to p < 0.01, *** corresponds to p < 0.001. Significant difference (+) from RT112 cells (+ p < 0.05, ++ p < 0.01). Cell number: (**A**) and (**C**) For 0.95 mg/mL, N = 9, 10, 7 respectively for J82, T24, RT112 cells. For 1.8 mg/mL, N = 8, 9, 7. For 4.5 mg/mL, N = 10, 13, 9. (**B**) For 0.95 mg/mL, N = 8, 9, 7 respectively for J82, T24, RT112 cells. For 1.8 mg/mL, N = 7, 8, 7. For 4.5 mg/mL, N = 8, 9, 9. (**D**) Graph representing the mean directionality vs. time for the three cancer cell lines at 0.95 mg/mL.

To determine how the metastatic potential of cancer cells is correlated with migration, we investigated the migration and effective speed of bladder cancer cells of increasing metastatic potential (RT112 < T24 < J82) in gels of different concentrations (0.95 mg/mL, 1.8 mg/mL, 4.5 mg/mL). Supplemental Movie S1 gives an idea of the motion of a T24 cell migrating in the low concentration collagen (0.95 mg/mL). Cell migration speeds (i.e. average of instantaneous speeds) and effective speeds (total distance traveled divided by total migration time) at various collagen concentrations are shown in Fig. 1A,C. The results reveal that the migration and effective speeds decrease with collagen concentration regardless of the metastatic potential. A strong decrease in migration speed is observed for J82 cells (65%), larger than in the case of T24 (50%) and RT112 cells (53%), as collagen concentration increases from 0.95 to 4.5 mg/mL, and similarly from 0.95 to 1.8 mg/mL (respective decrease of 56%, 35% and 38%). Regarding mean migration speed values, J82 cancer cells move with velocities in the range [7.3–20.9 μm/h], as compared to [7.5–14.9 μm/h] for T24 cells and [4.3–9.1 μm/h] for RT112 cells. A strong decrease is also observed in terms of effective speed of J82 cells as collagen concentration increases from 0.95 to 4.5 mg/mL (80%), larger than in the case of T24 (63%) and RT112 cells (57%).

Interestingly, these results also reveal that grade-2 cancer cells (RT112) migrate slower than J82 cells at the lowest collagen concentration. Differences in cell speed are also visible at high collagen content: the migration and effective speeds of grade-2 cancer cells are statistically lower than T24 cells. On the other hand, no significant differences were measured between T24 and J82 cell speeds whereas these two cell lines do not have the same cancer stage. Note that J82 cells display a broad range of speed values at low collagen concentration, and this will be discussed later.

To further characterize the migration behavior of cancer cells at different collagen concentrations, we studied the persistence of cell migration using the MSD power-law exponent $\alpha$ and diffusivity D (see “Materials and methods” section). Our data reveal that $\alpha$ and D are decreasing functions of the collagen concentration^34^. For the most invasive J82 cells (see Fig. 1B), $\alpha$ ranges from *super-diffusive* values (mean $\alpha \sim 1.39$) at 0.95 mg/mL to *random* ones (mean $\alpha \sim 1.09$) at 1.8 mg/mL, and *sub-diffusive* (mean $\alpha \sim 0.7$) at 4.5 mg/mL. Similar variations in power-law exponents were observed for T24 and RT112 cells but were not significant. Moreover, the diffusivity decreases by a factor of about 10 for J82 cells of about 4 for T24 and RT112 cells between 0.95 mg/mL and 4.5 mg/mL (see Table 1). Finally, at low collagen concentration, we found that RT112 migration behavior is random (mean $\alpha \sim 1.1$) as compared to the super-diffusive motions of T24 and J82 cells (respective means $\alpha \sim 1.37$ and 1.39) (Fig. 1B). A similar remark can be made in terms of mean directionality d(t) (see “Materials and methods”) : RT112 cells show an important decrease of the directionality in time as compared to T24 and J82 cells (Fig. 1D). Furthermore, these RT112 cell trajectories seem to reach limiting values around 0.2 whereas the T24 and J82 cell trajectories tend to 0.3–0.4. At this lowest collagen concentration, we also observed an effective speed of RT112 cells significantly lower than the J82 cell effective speed (Fig. 1C) confirming a very different behavior of J82 cells.

**Table 1:** Mean values of the diffusivity (in μm^2^) ± standard error of the mean.

Collagen concentration	Cell type	D (μm^2^)
0.95 mg/mL	J82	11.4 ± 3.8
	T24	8.3 ± 3.4
	RT112	2.8 ± 0.7
1.8 mg/mL	J82	3.4 ± 1.0
	T24	5.2 ± 1.7
	RT112	1.1 ± 0.2
4.5 mg/mL	J82	1.3 ± 0.3
	T24	1.9 ± 0.7
	RT112	0.7 ± 0.1

### Cancer cell morphology in 3D collagen matrices

**Figure 2:**
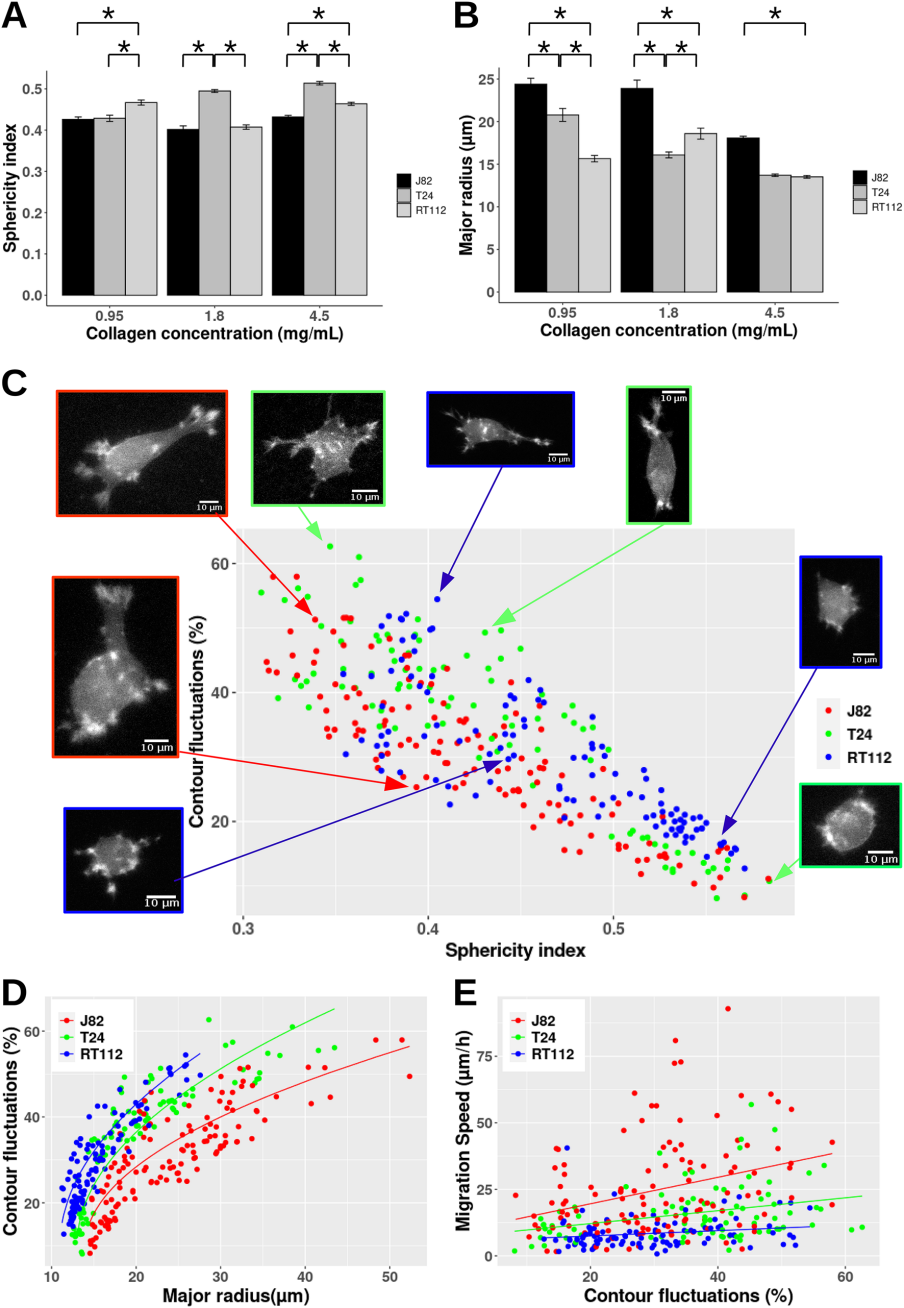
(**A**) Mean sphericity index ($\psi$) for different cancer cells (RT112, T24 and J82) in various collagen gels (0.95 mg/mL to 1.8–4.5 mg/mL). *p < 0.05 (Kruskal Wallis test). Error bars represent mean ± SEM. (**B**) Mean major radius (*R*) of different cancer cells at low, intermediate and high collagen concentrations. (**C**) Graph representing contour fluctuations ($\nu$) vs. sphericity index $\psi$ . Insets show various shapes used by cancer cells of various invasiveness (blue for RT112 cells, green for T24 cells, red for J82 cells). (**D**) graph of contour fluctuations ($\nu$) vs. major radius (*R*) in μm. Solid line: power fit of $\nu$ = A + B * (R − C)$^{1/2}$ with A = 10, 10, 10; B = 7.5, 10, 11; C = 14, 13, 11 respectively for J82, T24 and RT112 cells. (**E**) Migration speed (V) vs. contour fluctuations ($\nu$). Fits V = a*$\nu$ + b with a = 0.5, 0.25, 0.1 μm/h; b = 9.6, 7.3, 5.5 μm/h, for J82, T24 and RT112 cells.

To investigate the migration strategies used by the three cancer cell lines, we analysed the 3D cell morphology parameters (i.e. cell sphericity index, major radius) and introduced a new concept based on contour fluctuations. Figure 2A,B show means and SEM of sphericity indexes as well as major radii for the three cell lines at various concentrations. At low concentration, grade-3 cells (T24 and J82 cells) were found to have similar small values of sphericity index, significantly smaller than grade-2 cells (0.426 for J82 cells, 0.429 for T24 cells and 0.467 for RT112 cells). At intermediate (1.8 mg/mL) and high concentrations (4.5 mg/mL), T24 cells showed a higher sphericity (0.495 and 0.514 respectively) as compared to RT112 cells (0.407 and 0.464) and J82 cells (0.402 and 0.432). J82 cells showed a major radius significantly higher (24.4 μm, 23,9 μm and 18.1 μm at increasing concentrations) than T24 and RT112 cells for all concentrations. To summarise, the general morphology of cancer cells migrating in the high concentration gels is different from the one found at low concentration:

- J82 cells display a significantly smaller major radius compared to low collagen concentration, with similar sphericity index (around 0.43).
- T24 cells also show a significant smaller major radius compared to low collagen concentration but with a rounder shape (sphericity index of 0.514 significantly higher than 0.429).
- RT112 cells display a small major radius, with the same sphericity (index around 0.46).

To investigate further the diversity in cell phenotypes (i.e. morphologies) at low collagen concentration, the contour fluctuations *vs* sphericity index were plotted for each time (Fig. 2C). High values of the contour fluctuations are the signature of many cell protrusions (see insets in Fig. 2C) whereas low values (< 20%) correspond to migrating cells exhibiting few protrusions. Consistent with these observations, the graph shows that the lower the sphericity index, the higher the shape irregularity. The same trend was found for medium and high collagen concentrations (data not shown). We observed that cancer cells exhibit a range of sphericity indexes between 0.3 and 0.6, corresponding to morphologies going from highly polarized cells with many extending protrusions (contour fluctuations > 40%) to very round cell shapes with few short extensions such as small membrane blebs (contour fluctuations < 20%). However, a certain fraction of cancer cells with low sphericity index (index < 0.4) exhibits low values of contour fluctuations (< 40%). In particular, metastatic T24 and J82 cells display a larger range of sphericity indices (0.3 to 0.6) with important shape differences (contour fluctuations from 5 to 60%) whereas RT112 cells are restricted to a range of high sphericity indices (0.35 to 0.58) and smaller contour fluctuations (10 to 55%). To further characterize the effect of invasiveness on cell morphology, the relationship between contour fluctuations and major radius was plotted in Fig. 2D. This is an interesting criterion to determine cell invasiveness at low collagen concentration: J82 cells clearly exhibit the lowest fluctuation curve of all cell lines for a given size (Fig. 2D) revealing that large fluctuations are inefficient to enhance cell migration velocity (case of RT112 and T24 cells). This is also emphasized in Fig. 2E where the migration speed is plotted as a function of contour fuctuations. Straight lines are used as a fit and reveal that the correlation (or slope) is larger for J82 cells.

To summarize, these parameters reveal the round morphology or slightly elongated shape of RT112 cells with a small major radius, an elongated morphology for T24 and J82 cells and the ability of grade-3 cells to show a great diversity of shapes at low collagen concentration.

### Dynamic cell-induced 3D displacements

**Figure 3:**
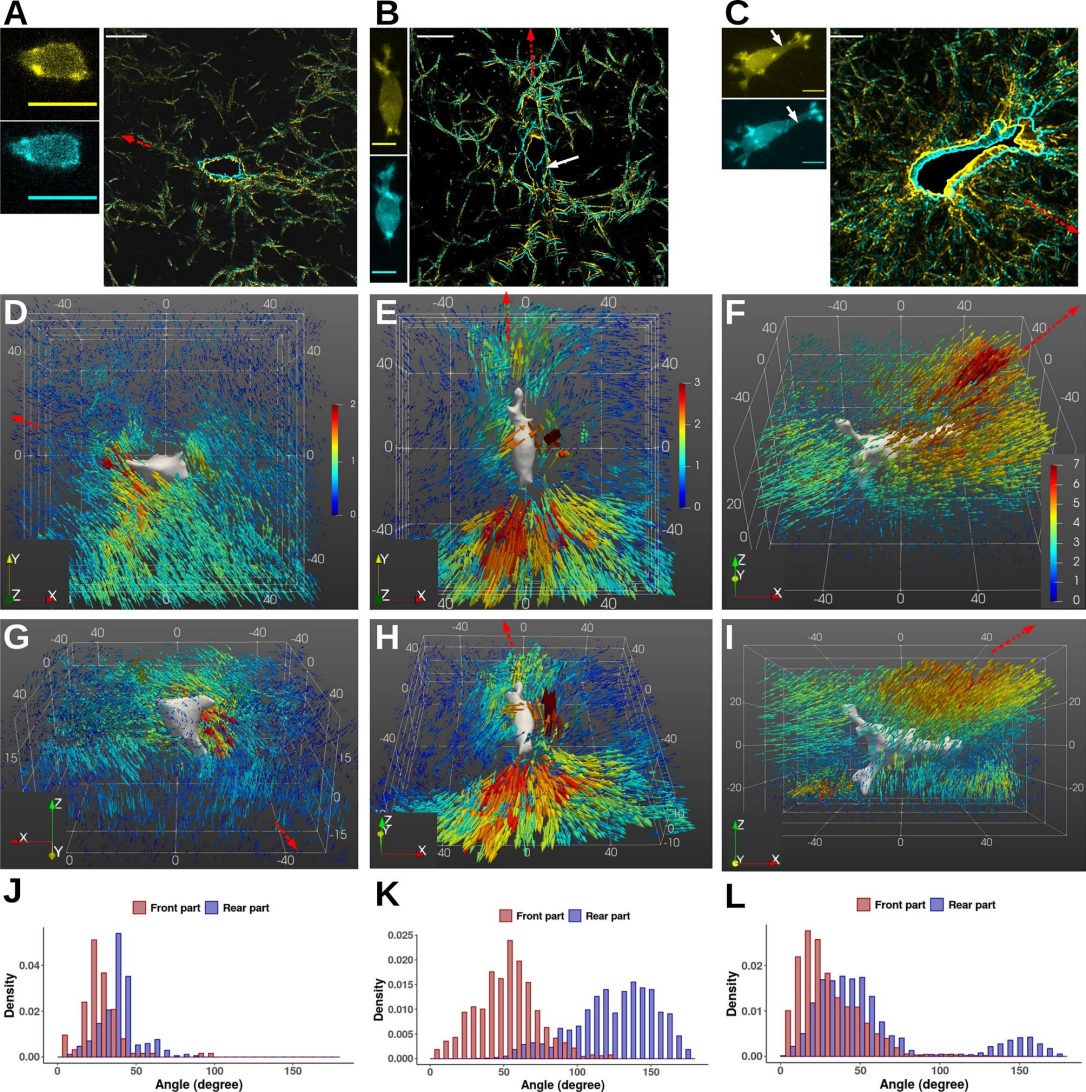
Boxes (**A**–**C**): Z-projections (left panels) of confocal fluorescence images of the actin cytoskeleton for three migrating bladder cancer cells (RT112, T24 and J82 respectively) embedded in a 0.95 mg/mL collagen gel, at 10 min time interval (first position in yellow, second one in cyan). Superposition of collagen fiber images (right panels) at these times, for one slice level in the image stack, with indication of cell contours. Scale bar = 20 μm. The white arrow indicates the rear of the T24 cell (**B**) or the long cylindrical protrusion displayed by the J82 cell (**C**). The red dotted arrow indicates the migration direction. Boxes (**D**–**I**): Corresponding 3D collagen fiber displacements around each migrating bladder cancer cells: RT112 (**D**,**G**), T24 (**E**,**H**), J82 (**F**,**I**). Two viewing angles have been selected. The initial 3D cell shape is shown in grey levels. The vector length and color indicate the displacement magnitude in μm. The x, y and z grids are in μm. The red dotted arrow indicates the migration direction. (**J**–**L**) Angle distributions of displacement vectors—with respect to the direction of migration—shown in red (resp. blue) for vectors located at the front (resp. at the rear) edge.

To study how cancer cells deform their environment, we determined the 3D displacements of collagen fibers induced by the migration of RT112, T24 and J82 cells. Figure  3A–C show the superposition of two successive reflectance confocal images obtained at 10 min time interval for each cell. The corresponding displacement fields around the cells are presented from two viewing angles (1rst angle in Fig.  3D–F, 2nd angle in Fig. 3G–I). The angle distributions of displacement vectors— relative to the direction of migration—are also shown in Fig. 3J–L.

RT112 cells show a quite round morphology (Fig.  3A,D) at both times with no clear cell shape polarization. F-actin is located at the cell periphery in the form of aggregates. A short extension is visible at the leading edge. The cell moves without important shape changes. Despite the round cell shape, the displacement field spans a relatively large zone (see Fig. 3D,G) with a maximal value of 2.6 μm. Large fiber movements (> 1.4 μm) are oriented preferentially between 10° and 65° at the front and at the rear (see Fig. 3J).

In Fig. 3B, the images of the T24 cell correspond to an elongated morphology with a protrusion at the leading edge and a contraction of the rear (see white arrow on the overlay and cell shapes on the left panel). The cell is polarized as seen from its actin distribution. Its motion corresponds to a highly asymmetric 3D displacement field with a maximal value of 3.6 μm (see Fig. 3E,H). Collagen displacements are seen far from the cell (as far as 50 μm from the cell surface). The largest displacements (> 1.2 μm) are along the cell major axis and important displacements localized on the lateral side are also visible. Moreover, collagen fibers move in opposite directions between the leading edge and the rear edge: displacement vector angles (see “Materials and methods”) lie in the range [10°–100°] at the front as compared to [60°–180°] at the rear (see Fig.  3E,K).

In the case of the J82 cell, the image overlay shows an elongated cell shape at both times (see Fig.  3C). These images together with the 3D-displacement field reveal a long cylindrical protrusion (see white arrow on the left panels of Fig. 3C,F,I). Note that the protrusion displays several actin-rich regions at the tip, and also blebs at the surface. The magnitude of the fiber displacements reaches important values (up to 7.1 μm) with vectors showing [0°–80°] orientation at the front and [0°–180°] at the rear (see Fig. 3L). The largest displacements (magnitude > 5 μm) are observed at the front along the migration axis and also at the rear with a mean orientation around 150° (see Fig. 3F,I). At the rear part, some displacements are also visible at long distances with a mean orientation around 50°.

Cell dynamics and collagen displacements can be summarized as follows:

- RT112 cells stay quite round with short extensions, while inducing extended fiber displacement fields with fibers moving in the direction of migration.
- The T24 cell is highly polarized with a protrusion inducing fiber movements away from the cell w.r.t. to the migration direction and a rear retraction generating fiber displacements directed away from the cell.
- The J82 cell motion induces collagen fiber movements in the direction of migration at the leading edge. Important and localized displacements are also visible at the rear.

These results are completed by a study of the collagen displacement dynamic in association with the cell behavior (see Supplemental Movies S2, S3 and S4). Movies show that T24 and J82 cells migrate with long or large protrusions. These cells show various displacement patterns and are able to push and pull on the collagen fibers. In contrast, the RT112 cell exhibits a slightly elongated shape with short extensions and only pulls on fibers located around protrusion/cell surface.

### Correlation between fiber displacements and the F-actin distribution

**Table 2:** Mean values of the largest fiber displacements (in μm) ± standard error of the mean.

Collagen concentration	Cell type	Mean displacement	p
0.95 mg/mL	J82	4.7 ± 0.6	
	T24	2.7 ± 0.2	**
	RT112	2.6 ± 0.2	**
1.8 mg/mL	J82	2.3 ± 0.1	
	T24	1.4 ± 0.4	***
	RT112	1.3 ± 0.1	***
4.5 mg/mL	J82	1.1 ± 0.1	
	T24	0.8 ± 0.1	***
	RT112	0.6 ± 0.1	*

Statistical significance p (in comparison to J82 cells) for each collagen concentration is also indicated: *p < 0.05, **p < 0.01, and ***p < 0.001.

**Figure 4:**
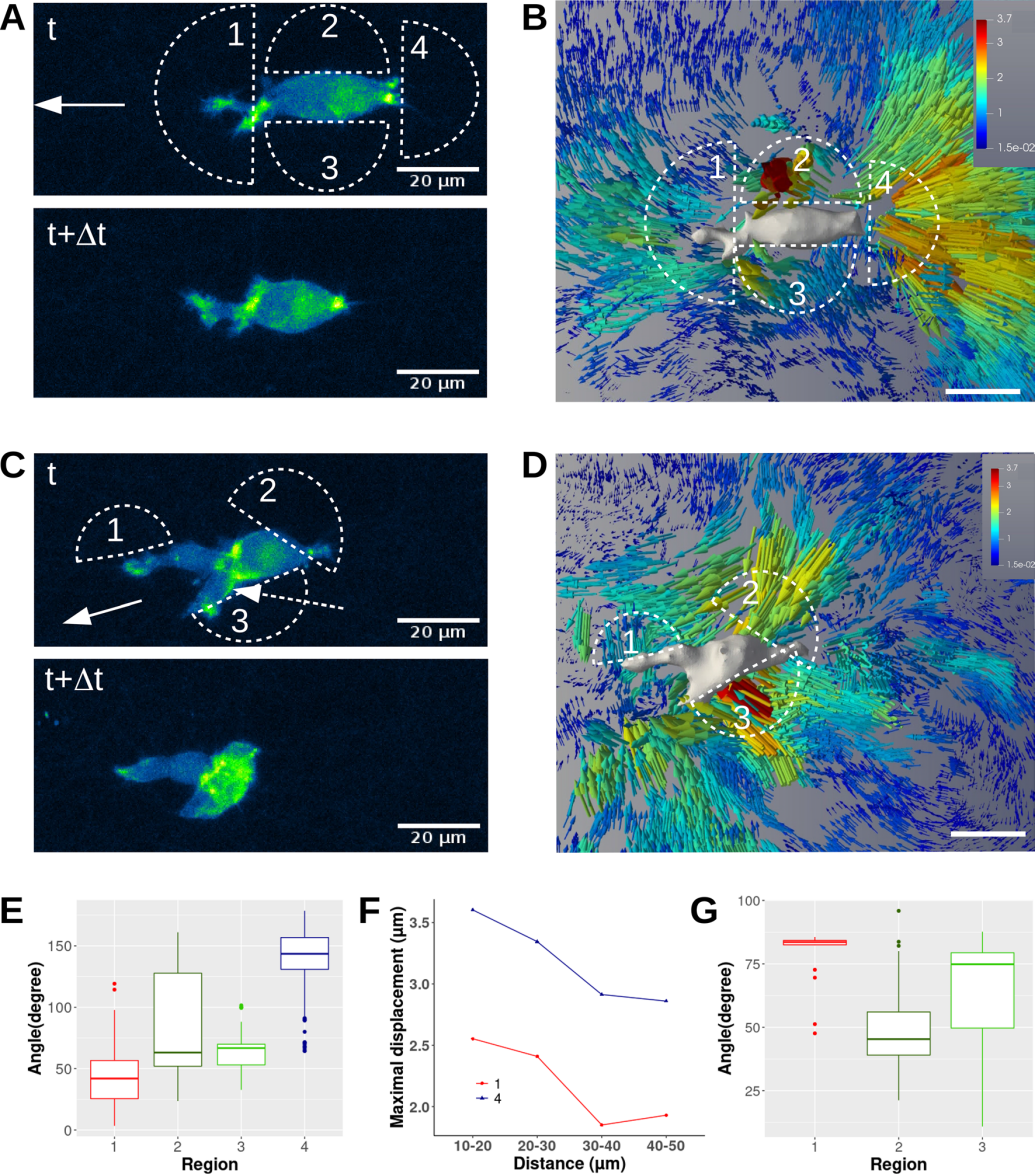
T24 cell migrating in 0.95 mg/mL collagen. (**A**) and (**C**) Two successive images of F-actin (projected Z-stack fluorescence). White half-circles indicate regions of interest. The white arrow indicates the direction of migration whereas the white dotted arrow indicates actin-rich regions inside the nucleus. (**B**) and (**D**) show the collagen fiber 3D-displacement field around the migrating cell. For more visibility, the initial 3D cell shape is shown in grey levels and the displacement field is shown for a half space. Vector lengths and colors indicate the displacement magnitude in μm. Scale bar = 20 μm. (**E**) and (**G**) Box and whisker plots of fiber displacement vector angles versus cell migration direction for regions of interest. (**F**) Maximum displacement amplitudes as a function of cell-fiber distance for the actin-rich regions 1 and 4 in (**A**).

In order to explore the role of metastatic potential on collagen deformations, we calculated the largest fiber displacements induced by the migration of bladder cancer cells for all times as well as the mean value of this maximal displacement for each cell line. This analysis is summarized in Table 2. At low collagen concentration (0.95 mg/mL), J82 cells—with the highest metastatic potential—generate significantly higher fiber displacements (mean value of 4.7 μm) as compared to T24 (2.7 μm) and RT112 cells (2.6 μm). Moreover, the magnitude of the largest displacements decreases significantly when collagen concentration increases for the three cell lines (see Table 2). Note that J82 cells show the highest value of displacement regardless of the collagen concentration.

Furthermore, to relate the largest fiber-displacements to the presence of high actin-density in the cytoskeleton, we compared the magnitudes and directions of displacement vectors in different regions. This analysis is shown in Fig.  4 for two migration stages of a T24 cell at low collagen concentration (0.95mg/mL) (image #1 and image # 5 of Supplemental Movie S3). For the first stage, the cell shows a highly polarized morphology at times t and t + ${\Delta }$t (Fig.  4A). We notice actin-rich regions at leading and rear edges for both times and actin speckles appear along the cell body (Fig. 4A). The cell-induced displacement field during ${\Delta }$t ($=10\,$min) exhibits large displacements located in regions 1 and 4 ($\sim$ 2 μm in region 1 at the front, $\sim$ 2.8 μm in region 4 at the rear, Fig.  4B). A study of the vector orientation with respect to the direction of migration showed a mean angle on the order of 42° at the leading edge (region 1) whereas the movement of fibers at the rear (region 4) is opposed to migration (mean angle 141°, Fig. 4E). This is consistent with the displacement field (Fig.  4B) showing vectors directed away from the actin zone at the front and back (Fig.  4A,B). This pattern corresponds to the progression of the leading extension associated with the cell rear-contraction (see Fig.  4A). Note that lateral sides (regions 2 and 3) correspond to high-displacement vectors (Fig.  4B) but these are very localized and parallel to the cell surface. The mean angle in regions 2 and 3 is about 82° and 63° respectively. As anticipated, we note that the amplitude of the maximal displacements decreases as a function of cell-to-fiber distance for the actin-rich regions (regions 1 and 4 in Fig.  4F), but this decrease is not observed for the other side areas (regions 2 and 3).

During the second stage (Fig.  4C), the cell is polarized and exhibits two cylindrical protrusions. The actin-rich regions (aggregates/filaments, see dotted arrow in Fig.  4C) are mainly localized in front of the nucleus and small actin spots are also visible at the end of protrusions. Surprisingly, in the nucleus periphery, we observe high displacements oriented towards the nucleus (regions 2 and 3 in Fig.  4C,D). Indeed, displacement angles take values between 49° and 65° with respect to the direction of migration (Fig.  4G). We also pay attention to the large movements of collagen fibers around the cylindrical protrusion and analyze their orientation (regions 1 in Fig.  4G). In this region, the mean angle is 79°. Thus, very localized and perpendicular fiber movements can be seen in the vicinity of protrusions. As before, close to the large actin-rich regions (2 and 3), we observe large displacements of collagen fibers with their amplitude decreasing with cell-fiber distance (Fig.  4D). Taken together, these results clearly indicate that high fiber displacements are correlated with the presence of actin-rich regions within the cell. The displacement vectors are either directed away from or toward these large actin-rich areas. In the vicinity of these regions, displacement fields can be large and decrease as a function of cell-fiber distance.

This correlation between actin density and displacement fields can also be found in the collagen displacement movies (see Supplemental Movies S2, S3 and S4). It is enhanced for T24 and J82 in comparison with RT112 cells.

**Figure 5:**
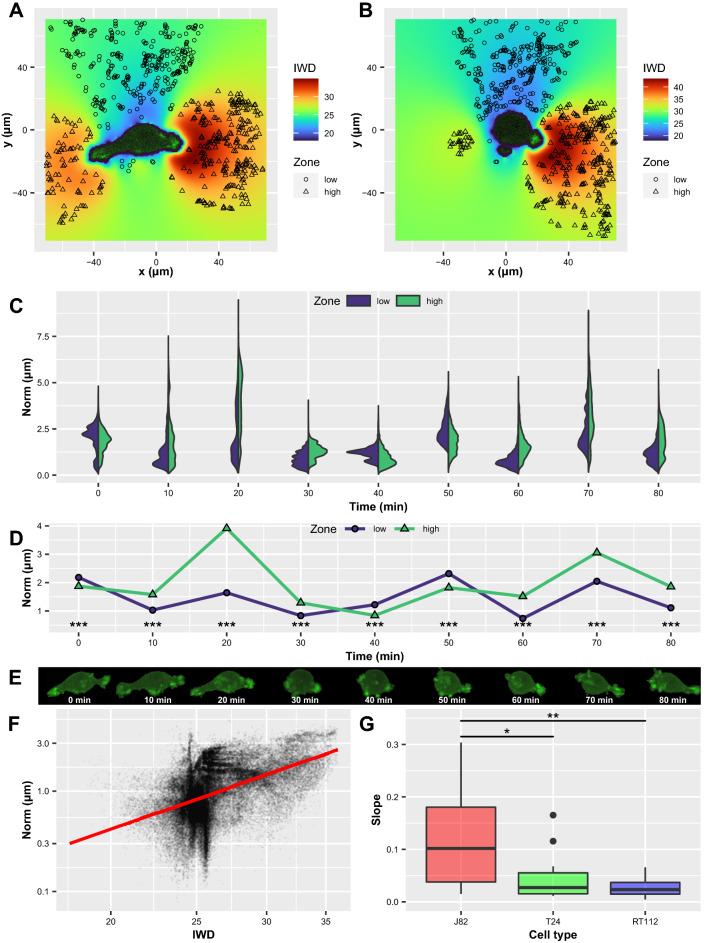
Correlation between actin intensities and displacement magnitudes (J82 cell). (**A**,**B**) Values of IWDs are shown at two different times, 20 min, and 30 min. Symbols represent low and high IWDs, respectively [0–25%] and [75–100%] ranges. (**C**) Violin plots of the displacement norm distributions corresponding to low (blue) and high (green) IWD values in time. (**D**) Median of the norms (low and high IWDs) vs. time, with significant difference from Kruskal-Wallis test. (**E**) Corresponding cell shapes. (**F**) Displacement norms vs. weighted actin intensities (IWD) for t = 20 min. The red line is the regression curve. (**G**) Slopes of regression curves at times for which Norm$_{high}$/Norm$_{low}$> 1, for the three cell types. Significance of Kruskal-Wallis test, followed by Dunn’s test with Bonferroni adjustment.

Finally, to prove the reliability of this previous arbitrary choice, we carried out a systematic analysis to obtain correlations between actin intensites and displacement magnitudes (or norms) for all cases. Fig.  5 explains the procedure used. For example, a J82 cell is followed in time. Here, two time steps are shown (Fig.  5A, t = 20 min and Fig.  5B, t = 30 min) and Intensity Weighted Distances (IWDs, as defined in “Materials and methods” section) are visualized. IWDs are splitted into two sub-ranges corresponding to low and high values (respectively [0–25%] and [75–100%] ranges). This cell is elongated at t = 20 min, then retracts at t = 30 min. Therefore we expect strong displacements to be correlated with intense actin spots at time t = 20 min. To check this, the time-evolution of the corresponding displacement norms (low and high domains) is described in Fig.  5C,D (violin plot of displacement norm distributions and median values). The cell behaves differently in time. The corresponding actin spots can also be observed in Fig.  5E. A selection of the more interesting times can be done using the criterion Norm$_{high}$/Norm$_{low}$>1. Doing this allows to present a cloud of data points (Fig.  5F) for vector norm vs. actin intensity, for example here at t = 20 min. The behavior, as shown by the linear regression (in red) gives a positive slope. All cases for the 11 cells studied and a total of 49 times gave positive slopes. These slopes are compared for the three cell types (J82, T24 and RT112) in Fig.  5G and show differences, with a larger significant slope in the case of the most invasive J82 cells.

## Discussion

The mechanisms of cell migration have been widely studied but were very often limited to 2D motion on various substrates simplifying cell-substrate interactions, cell morphology and obviously cytoskeleton dynamics. Recently, there has been considerable interest to investigate the cell motion in 3D matrices that better mimick the physiological environment. These results highlight the biophysical role of the matrix microarchitecture on cell behavior^35^, the contribution of cell contractility, the role of fiber stiffness in 3D migration^36^, the role of actin bundles and integrins in force transmission to the surrounding environment^30^. Moreover, other studies emphasize a cell plasticity mechanism (mesenchymal-to-amoeboid transition) in cell migration favoring tumor dissemination and metastasis^22,37^. Cancer cells can use different modes of migration depending, among others, on the physical properties of the ECM^19,38^. However, much of our knowledge about the migration in 3D environments comes from studies involving cells from different cancer types. Moreover, these studies do not combine simultaneously spatial and temporal analysis of cell behavior and cell-induced environment deformations.

In this study, we investigated how the malignancy of bladder cancer cells is related to their migration strategy in 3D collagen matrices mimicking physiological environments. We used three bladder cancer cell lines with different degrees of metastatic potential: RT112, T24 and J82 cells. The choice of these cells comes from previous studies, where various properties associated with their malignancy, such as adhesion and rheology, were reported^32,39,40^. RT112 cancer cells are moderately differentiated (grade 2), whereas T24 and J82 cancer cells are poorly differentiated (grade 3) and the J82 cell line has higher metastatic potential (see “Materials and methods”). In addition, these cancer cells were used before as models for molecular classification and exhibit different mTOR-associated gene expression which is relevant for chemotherapeutic strategy^41^. They express $\alpha$5 and $\beta$1 integrin subunits which are known to play a role in cell invasion by enhancing transmission and generation of contractile forces^42,43^.

To avoid collagen degradation during cell migration, we limited experiments to a maximum time of 100 min, which restricts the MMP activation, therefore enzymatic degradation of matrices. To test if the cancer cell motility response to increasing matrix rigidity is malignancy dependent, we measured migration speeds at different collagen concentrations, 0.95, 1.8 and 4.5 mg/mL, corresponding to respective Young moduli E $\sim$ 20, 250 and 1400 Pa^13^. We found values of migration velocities in agreement with recent data obtained in 3D rat tail collagen gels^36,37,44^. It should be noted that Fraley et al.^45^ obtained higher values of speeds as they conduced cell motility studies up to 16.5 h and they noticed that speed values decrease after MMP inhibitor treatment. As already noticed by Wolf et al.^37^, we also observed that increasing the collagen concentration induced a decrease in the migration speed, regardless of the metastatic potential of bladder cancer cells (see Fig. 1A). This result suggests that an increase in stiffness from 10 Pa to 1000 Pa has a significant effect on cell migration speed regardless of bladder cancer cell lines because of the limited space available due to smaller matrix pore size. Nevertheless the impact is more important for J82 cells: the relative decrease of J82 mean speed (65%) is more important than T24 (50%) and RT112 cells (53%) when collagen concentration increased from 0.95 to 4.5 mg/mL. A recent study demonstrated that the migration velocity of partially- or fully-transformed breast cells decreases when collagen density increases from 2 mg/mL to 4 mg/mL^26^, this effect being more important for partially transformed cells. Our findings do not show the same trend as the migration speed decreases similarly for the three cell lines ($\sim$ 20% from 1.5 to 4.5mg/mL). Possible explanations for this discrepancy could be related to cell type. Indeed, Baker et al.^26^ examined breast cancer cell progression series of lines established from a non cancerous human line whereas we used bladder cells lines established from human bladder cell carcinomas.

In addition, we also identified that collagen concentration plays a significant role for the movement persistence of J82 cells: the analysis of the mean square displacement (MSD) showed that the power law exponent (Fig. 1B) significantly decreased when increasing collagen concentration for grade-3 J82 cells whereas no significant reduction was measured for the other cell lines. Moreover, this analysis highlighted a super-diffusive motion at low concentrations ($\alpha$ $\sim$ 1.39) and a sub-diffusive one at larger concentrations ($\alpha$ $\sim$ 0.7). These results were confirmed by higher values of diffusivities and mean directionalities for grade-3 cells (T24 and J82 cells) in comparison of RT112 cells (see Table 1 and Fig. 1D). We concluded that grade-3 cell cells move more efficiently as compared to RT112 cells. Moreover, J82 cells exhibit the most effective migration. All these findings go along with morphological changes: an increase in collagen concentration (0.95 to 4.5 mg/mL) induces a decrease of the major radius of J82 cells (Fig. 2B) and a reduction of contour fluctuations (30.7 ± 1.0% to 24.0 ± 1.0%) which is the signature of the number of extensions. In summary, the impact of collagen stiffness increase is more important for malignant J82 cells in terms of speed reduction, movement persistence and diffusivity. These results also suggest that matrix rigidity sensing is of utmost importance for malignant tumor cell motion, and that the response of grade-3 cells is characterized by changes of the major radius and contour fluctuations allowing cells to move more easily in less dense matrices.

Since J82 and T24 cancer cells migrate faster than RT112 cells in 0.95 mg/mL collagen, and the same for T24 vs. RT112 at 4.5 mg/mL, we compared the morphological parameters of the bladder cancer cell lines. At 0.95 mg/mL, grade-3 cells (T24 and J82) displayed mean sphericity indexes significantly lower than grade-2 cells (RT112). Moreover, by looking at the contour fluctuations as a function of sphericity index, we observed that grade-3 cells exhibited a larger range of sphericity indexes and contour fluctuations than grade-2 cells. Therefore grade-3 cells show a great diversity of shapes (from highly elongated to very round ones) with variable numbers and types of protrusions (Fig.  2C). This diversity of shapes and protrusions is coherent with the recent study of Eddy *et al.*^24^. However, J82 cells have a larger size as compared to other cells but exhibit the lowest shape fluctuations for a given major radius. These results suggest that a combination of low sphericity index and ability of cells to display a large range of contour fluctuations is optimal for migration in low collagen concentration. But the most interesting criteria to recognize the migration of a given cancer cell is by comparing its contour fluctuations at a given size (see Fig.  2D) or look at the migration speed as a function of contour fluctuations (Fig.  2E). These two graphs definitely exhibit different modes of migration used by invasive cells.

The less invasive cells (RT112 cells) showed a round or slightly elongated shape with short extensions or fine extensions or membrane blebs (see inserts in Fig.  2C and Movie S2). The analysis of the displacement field indicates that RT112 cells can move without important shape changes (Movie S2) and no clear shape polarization. RT112 cells show sparse fiber movements directed in the migration direction along the cell surface (Fig. 3D and images #2, #3 and #5 Movie S2). Furthermore, we observed that high-density F-actin spots are visible at the cell periphery but not distributed isotropically (see Fig.  3A and Movie S2). This could signify that asymmetric actin accumulations at the cell periphery are needed to achieve directional migration and are consistent with previous studies about actomyosin contractility in amoeboid mode^46^. We also noticed that RT112 cells showing a relatively wide and short protrusion can generate fiber displacements around the edge of protrusions (image #1 Movie S2). Notably, the cell produces scattered pulling movements on fibers. These results are consistent with an amoeboid cell behavior whose migration is independent of cell matrix adhesions^47^.

Previous studies reported that highly polarized cancer cells in 3D matrices induce large displacements near the protrusive tip^27^ and that forces are used almost exclusively to pull collagen fibers towards the cell^29,48,49^. The results presented here showed that the migration of an elongated T24 cell can exhibit a series of various displacement patterns (see Supplemental Movie S3). First, protrusions push fibers at the front whereas the cell retracts at the rear. This creates relaxation of fibers away from the cell (Fig.  3E,H and first image of Movie S3). In the next step, elongation of protrusions and adhesion/anchoring to collagen fibers results in a pulling force in the polarization direction as well as on the lateral sides (image #3 of Movie S3). To our knowledge, these are the first time-dependent measurements of displacement fields showing that bladder cancer cells use a mesenchymal mode of migration thanks to protrusions, attachment, contraction and detachment, as in 2D^14^. Obviously, since our study used 10 min time lags, we observed the different mechanims at the same time. The formation of a 3D-lamellipodium at the leading edge generating a pushing force in the direction of migration and the relaxation of the collagen fibers at the rear (after the rear detachment) are visible in image #1 of Movie S3. Interestingly, a clear difference appears between 2D and 3D migration: during the contractile phase, several actin fibers are localized in front of the nucleus, generating a pulling force. The amplitude of these contractile displacements decreases with cell-surface distance (Fig.  4D). We also noticed the same decrease of the pushing displacement at the front while fibers relax at the rear, close to the large actin-rich structures (Fig.  4F).

The migration data showed that invasive J82 cells are elongated and migrate using long cylindrical protrusions similar to a 3D-lamellipodium with high concentration of actin at the tip, with rapid membrane blebbing along the sides (Fig. 3F,I and Supplemental Movie S4)^50^. J82 cells induce large displacement fields (maximum value of 7.2 μm) and the membrane is very dynamic (see Table 2 and Supplemental Movie S4). These results agree with recent studies describing mesenchymal-like phenotype of T24 and J82 cells^51^. Deb et al. showed that the integrin pathway was significantly enriched and some major proteins regulating actin-polymerisation activity, cell motility and invasion are hyperphosphorylated for these two cell lines. Displacement fields for invasive J82 cells seem to be different from T24 cell: the cell pushes at the front whereas collagen fibers are pulled at the back of the nucleus to move the cell forward, while protrusions are growing (Fig. 3F,I and images #1, #2 and #5 in Movie S4). Then, cell movement produces relaxation of those fibers located near the end of protrusions (where actin-rich areas are visible, images #2, #3 and #4 in Movie S4). Note that the migration of the J82 cell gives rise to large displacements scattered in all directions (Movie S4). These fiber displacements are the largest in comparison to T24 and RT112 cells, regardless of collagen content. This suggests that J82 cells generate stronger forces.

This was finally assessed by a systematic analysis of the correlations between actin intensites (as obtained with the Intensity Weighted Distance, the so-called IWD) and corresponding displacement magnitudes. As shown in Fig. 5A,B, cells develop a variety of mechanisms. After selecting particular times when cells are polarized or exhibit a loss of symmetry (Fig.  5C,D), we studied the correlation between displacement norm and actin intensity at these times, and showed positive correlation (Fig.  5F). Therefore larger displacements are always found in the regions close to the actin-rich cell domains. In addition, this correlation is significantly stronger (Fig.  5G) for the more invasive J82 cells.

## Conclusions

Cell migration in 3D matrices is a complex mechanism: the spatial deformation of the environment is not uniform and follows steps depending of cell phenotype. It is crucial to understand this spatio-temporal process in the case of cancer cells. Using 3D time-lapse imaging on a confocal microscope and a phase correlation method, we followed fibers deformations, as well as cell morphology and live-actin distribution during cell movement. Our findings agree with a recent study describing the mesenchymal-like phenotype of T24 and J82 cells^51^. In addition, our study revealed that grade-3 cells (T24 and J82 cells) were characterized by a great diversity of shapes in comparison to grade-2 cells (RT112 cells). Furthermore, we showed that the small shape fluctuations of highly invasive cells (J82) enable their migration in fibrous matrices with low collagen content. Our results also revealed that J82 cells produce the largest fiber displacements whatever the matrix rigidity. The detailed analysis of cell movements enhanced the fact that bladder cancer cells were also able to exhibit different phenotypes (mesenchymal and amoeboid) according to their metastatic potential. The different spatio-temporal mechanisms were emphasized and we showed that cells can push and/or pull collagen fibers in order to deform collagen efficiently. The actin-rich regions were found to be correlated with the largest displacement fields and the correlation is enhanced in the case of grade-3 cells.

## Materials and methods

### Cell culture

Three epithelial bladder cancer cell lines, representing increasing malignancy states of progression, were used, RT112, T24 and J82 (ATCC, Rockville, MD). RT112 cells show an intermediate grade-2 and a stage Ta, i.e. these cells are moderately differentiated and have a small metastatic potential. T24 and J82 cells show a higher grade (grade-3) compared to RT112 cells and exhibit higher stages (T2–T3 and T3 respectively) i.e. they are poorly differentiated. J82 cells have the highest metastatic potential^32,52^.

Cancer cells were cultured in RPMI 1640 complete medium (Gibco, Saint Aubin, France) supplemented with 10% fetal bovine serum (FBS) and 1% penicillin-streptomycin. The cell lines were stably transfected with the LifeAct GFP plasmid to stain F-actin^53^. Cultures were grown at 37 °C in 5% CO_2_ atmosphere. Cells were let to grow until 50% confluency was reached. Then they were detached from culture dishes using trypsin-EDTA (0.25%), were re-suspended at $2 \times 10^{5}$ cells/mL concentration in culture medium, and finally seeded in collagen gels.

### Collagen gel sample preparation

Gels were prepared from a rat tail collagen type I solution (Corning, New-York, USA) in Nunc LabTek II chambered coverglass 8 wells (Gibco, Saint Aubin, France). In order for the collagen gel to strongly adhere to the bottom glass surface, LabTeks were first treated with sodium hydroxide (NaOH 0.1N) for 1 min, and allowed to dry. Then 3-Aminopropyl-trimethoxysilane (APTMS) was added for 10 min. After this treatment, LabTeks were cleaned 3 times with distilled water and dried. Finally, glutaraldehyde (0.5% in PBS) was added for 30 min, and after the solution was removed, LabTeks were rinced 3 times with distilled water and allowed to dry before adding the collagen solution. All the following steps were then carried out in ice (around 4 °C) to prevent polymerization of the collagen before adding it into the LabTeks. Solutions containing collagen (0.95, 1.8 and 4.5 mg/mL), RPMI medium supplemented with glutamine, FBS, antibiotics and fibronectin (10 μg/mL) were prepared. After adding NaOH (0.1 M) to reach a pH of 7.4, freshly harvested cancer cells were included in the collagen mixture to obtain a final concentration of 2 × 10^6^ cells/mL. The solution was poured into the LabTeks and transfered to an incubator for gel polymerization at 37 °C and 5% CO_2_ for 30 min.

### Live cell and collagen fibers imaging

The collagen fibers and the cell actin cytoskeleton were visualized simultaneously using the reflection and fluorescence imaging modes on a confocal microscope (Zeiss LSM710 model, Germany, equipped with a 40X water immersion objective, NA = 1.1). For this purpose, the 488 nm argon-laser wavelength was used to visualize the actin cytoskeleton of GFP-transfected cancer cells RT112, T24 and J82^52^, in combination with a 633 nm HeNe Laser for the reflectance imaging of collagen fibers. The microscope set-up used a 80/20 beam splitter that directed 20% of the reflected light by the sample to the detector^13^. A specific chamber (37 °C, 5% CO_2_) was mounted on the microscope stage in order to maintain cells in physiological conditions. Only isolated cells were investigated.

To construct a 3D image of migrating cells in a collagen matrix, we acquired Z-stacks of fluorescent/reflection images. The number of optical slices was different depending on cell acquisitions but the distance between two optical slices remained equal to 0.77 μm for all acquisitions. Image sizes were 512 × 512 × 128 pixels, with corresponding voxels 0.20 μm × 0.20 μm × 0.77 μm (or 0.32 μm × 0.32 μm × 0.77 μm for zooming purposes). To study the migration of cancer cells, the total acquisition time was around 2 h and stacks were acquired every 10 min.

### 3D migration characterization

To quantify 3D migration^25^, the velocity of cancer cells was calculated by tracking their center of geometry, using a home-made plugin under ImageJ. We used the green channel to locate the cell, and a threshold to determine its contour. The voxels inside the contour were kept, the volume was calculated and the center of geometry was determined precisely. This is the best way to obtain accurate data, as compared to other tracking methods such as bounding box center or ellipse fitting. Its position vector was written as $\mathbf{r}$(t), using the initial cell position as the origin. From the position vector, the instantaneous migration velocities were obtained and averaged in time^34^. Cells were classified as migrating cells if the center of geometry moved with a velocity higher than 3 μm/h^54^. The effective speed was calculated as the ratio of the distance traveled (from the initial to final positions of the cells) over total migration time. To evaluate the ability of the cancer cell to move in a complex Extra-Cellular Matrix (ECM), we calculated the mean square displacement (MSD) from the cell trajectories as follows:

1 $$\begin{aligned} MSD(t) = \left\langle \left| \mathbf{r} (t + \tau ) -\mathbf{r}(\tau ) \right| ^2\right\rangle _{\tau } \end{aligned}$$

where $\tau$ is the lag time, and the brackets represent the sum of possible changes as $\tau$ varies from 0 to the longest time T. The MSD is usually characterized using a power-law relationship of the type MSD(t) = D$(t/t_0)^\alpha$ where the exponent $\alpha$ represents the persistence of migration^55^, D is the diffusivity and $t_0$ is the lag time. $\alpha = 1$ corresponds to a ramdomly migrating cell while a value of 2 indicates a ballistically migrating cell. For the intermediate cases, $0<\alpha <1$ corresponds to a sub-diffusive motion whereas $1<\alpha < 2$ corresponds to a super-diffusive migration. The MSD power-law exponent was computed from the cell trajectories recorded during 2 h. When the MSD was not linear at large time lags, we computed the power-law exponent on a limited linear domain (fit on the first 60 min, to avoid the role of MMPs that could affect cell motion). Directionality d(t) was calculated from the ratio of the euclidean distance $e_d$(t) between the starting and current positions divided by the real distance $r_d$(t) travelled by the cell at time t : d(t) = $\frac{e_d(t)}{r_d(t)}$.

### Analysis of 3D-cell morphology

To investigate the morphology of migrating cancer cells in 3D-collagen matrices, we analysed their shape using a set of 3D-geometrical parameters. To capture this morphology with precision, we acquired confocal Z-stacks with 0.77 μm thickness slices. Then we applied the following procedure for all cells : a 3D gaussian blur ($\sigma _x$ = $\sigma _y$ = 1 in x, y and $\sigma _z$ = 2.6 in z), a median filter and thresholding using the OTSU algorithm. The cell major radius (*R*), obtained by fitting the cell contour by an ellipsoid, was defined as the length of the major axis of the ellipsoid. The sphericity $\psi$ (ranging from 0 to 1) was defined as the ratio of the surface area of a sphere (with the same volume $V_{p}$ as the cell) to the cell surface $A_{p}$.

2 $$\begin{aligned} \psi = \frac{\pi ^{1/3}(6V_p)^{2/3}}{A_p} \end{aligned}$$

$\psi$ equals 1 for a sphere and is close to 0 for a very elongated cell. Finally, we calculated the variation coefficient $\nu$ of the distance from the cell surface to its center, *i.e.* the ratio of the standard deviation (*SD*) to the mean distance ($d_m$): $\nu =\frac{SD}{d_m}$. This ratio of contour fluctuations (in %) was evaluated in order to indicate the cell shape irregularity (ranging from 5$\%$ to about 60% in this study).

### 3D displacement of collagen fibers

The 3D displacement of collagen fibers induced by the migrating cells was determined using a home-made phase correlation algorithm^33^ applied to the reflection image stacks obtained in time. The basic idea is to correlate only the positions of these fibers that are bright enough. Then we obtain incremental 3D displacements between times *t* and $t+\Delta t$. The phase-only correlation (POC) was used and reads as:

3 $$\begin{aligned} r({{\varvec{x}}} )={\mathscr {F}}^{-1}\left( \frac{I_1^*(\omega )*I_2(\omega )}{\left| I_1^*(\omega )*I_2(\omega )\right| } \right) \end{aligned}$$

where $i_1({{\varvec{x}}})$ is the image intensity at time *t*, $I_{1}(\omega )$ the Fourier transform of $i_1$, $I_{1}(\omega )={\mathscr {F}}(i_1({{\varvec{x}}}))$, ${\mathscr {F}}^{-1}$ is the inverse Fourier transform, $\omega$ the angular frequency, ${{\varvec{x}}}$ the position vector, and $I_{1}^{*}(\omega )$ denotes the complex conjugate of $I_{1}(\omega )$. The same applies for $i_2$ and $I_2$ at time $t+\Delta t$. The most noticeable property of the POC function is that it exhibits a unique sharp peak when the signal $i_{2}$ is a shifted version of $i_{1}$. Using this property, we obtain the required displacement ${{\varvec{u}}}$ in voxels, by locating the position of the peak of r(***x***):

4 $$\begin{aligned} {{\varvec{u}}} = \mathop {{{\,\mathrm{arg\,max}\,}}}\limits _{x} (r({{\varvec{x}}})). \end{aligned}$$

The discrete displacement was calculated using two passes on a 512 × 512 × 128 pixels image. We started with a large window (64 × 64 × 16) located around the point of interest, and obtained a first estimate $\varvec{u}$ of the displacement using phase correlation as described above, where the displacement is expressed in voxels. Then a smaller window (16 × 16 × 16) was used to estimate the extra displacement $\delta \varvec{u}$ using sub-pixel resolution as explained previously^56^. The final displacement $\varvec{u}_{sub}$ is the sum of the two displacements $\varvec{u}_{sub}=\varvec{u} + \delta \varvec{u}$. Window parameters have been optimized and correspond to the ones given above. Note that de-noising was used by removing data with a small correlation coefficient, and other irrelevant displacement vectors showing very large differences with their neighbors^33^.

### Correlation between actin intensity and displacements

In order to capture precisely the relationship between actin intensities (or spots) with the displacements, we developed a systematic analysis to relate the displacement at position **x** with the closest actin region (within the cell at positions **x**$_c$). Different methods were used but the most satisfactory one consists in calculating the weighted actin density at the required displacement position **x**, i.e. the so-called IWD (Intensity Weighted Distance) :

5 $$\begin{aligned} IWD(\mathbf{x })= \sum _{c}^{} I(\mathbf{x} _{c} ) w(\mathbf{x}-\mathbf{x} _{c} )/\sum w(\mathbf{x}-\mathbf{x} _{c}) \end{aligned}$$

where I($\mathbf{x} _{c}$) is the actin intensity at any voxel within the cell (the sum is on the cell voxels), and $w(\mathbf{x }-\mathbf{x} _{c})=1/\Vert {\mathbf{x }-\mathbf{x} _{c}} \Vert ^{\alpha }$ is a weighting function based on the Euclidian distance between **x** and $\mathbf{x} _{c}$. $\alpha$ = 8 was found to be well adapted, indeed the weight should be stronger for the closest areas. This gives the actin weight at the point of application of the displacement.

To go further, we split the IWD regions (colored ones) into two major regions corresponding to lower [0–25%] and upper IWD quartiles [75–100%] for simplicity. These two regions are named $\ll$high$\gg$ (triangles) and $\ll$low$\gg$ (circles). The corresponding magnitudes (norms) of displacements have to be correlated to the values of IWD. They are plotted in time.

### Statistics

Statistical analyses were performed using ANOVA. p < 0.05 was considered to be significantly different (*), ** corresponded to p < 0.01, *** corresponded to p < 0.001. All error bars were calculated using the standard error of the mean (SEM). When normality tests were not verified, statistical analyses were calculated using the Kruskal-Wallis test (morphological parameters and effective speed). p values < 0.05 were considered statistically significant.

## Electronic supplementary material

Supplementary Figure S1.

Supplementary Legends.

Supplementary Movie S1.

Supplementary Movie S2.

Supplementary Movie S3.

Supplementary Movie S4.
