# Supplementary material for: Efficient deformation mechanisms enable invasive cancer cells to migrate faster in 3D collagen networks
Source: Sci Rep. 2022 May 12;12:7867. doi: 10.1038/s41598-022-11581-2 (PMC9098560; doi:10.1038/s41598-022-11581-2)
Supplement: Supplementary file 1 — Supplementary Figure S1. [file 41598_2022_11581_MOESM1_ESM.pdf]

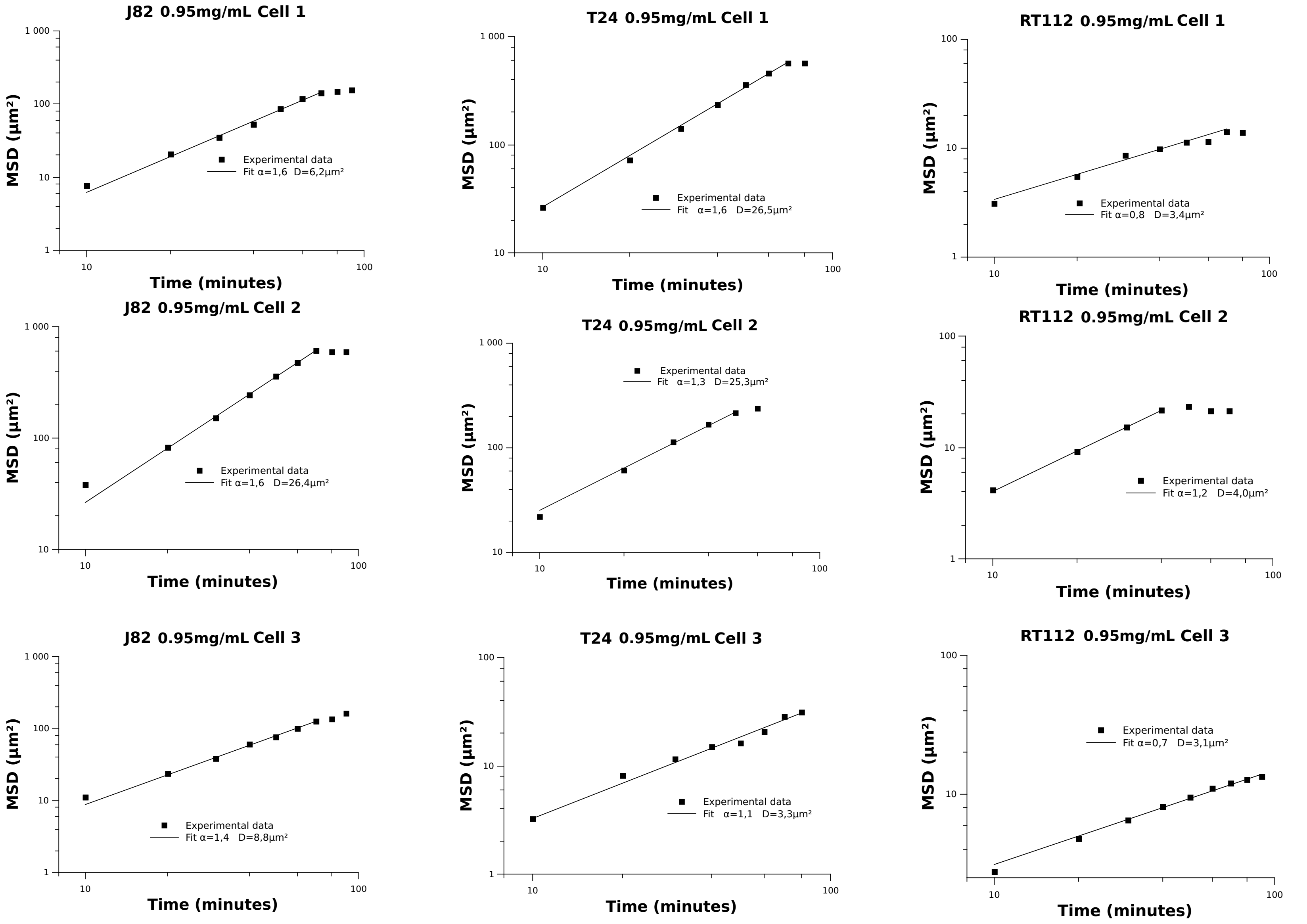

J82 0.95mg/mL Cell 1
MSD (µm²)
Time (minutes)
Experimental data
Fit α=1,6 D=6,2µm²
T24 0.95mg/mL Cell 1
MSD (µm²)
Time (minutes)
Experimental data
Fit α=1,6 D=26,5µm²
RT112 0.95mg/mL Cell 1
MSD (µm²)
Time (minutes)
Experimental data
Fit α=0,8 D=3,4µm²
J82 0.95mg/mL Cell 2
MSD (µm²)
Time (minutes)
Experimental data
Fit α=1,6 D=26,4µm²
T24 0.95mg/mL Cell 2
MSD (µm²)
Time (minutes)
Experimental data
Fit α=1,3 D=25,3µm²
RT112 0.95mg/mL Cell 2
MSD (µm²)
Time (minutes)
Experimental data
Fit α=1,2 D=4,0µm²
J82 0.95mg/mL Cell 3
MSD (µm²)
Time (minutes)
Experimental data
Fit α=1,4 D=8,8µm²
T24 0.95mg/mL Cell 3
MSD (µm²)
Time (minutes)
Experimental data
Fit α=1,1 D=3,3µm²
RT112 0.95mg/mL Cell 3
MSD (µm²)
Time (minutes)
Experimental data
Fit α=0,7 D=3,1µm²

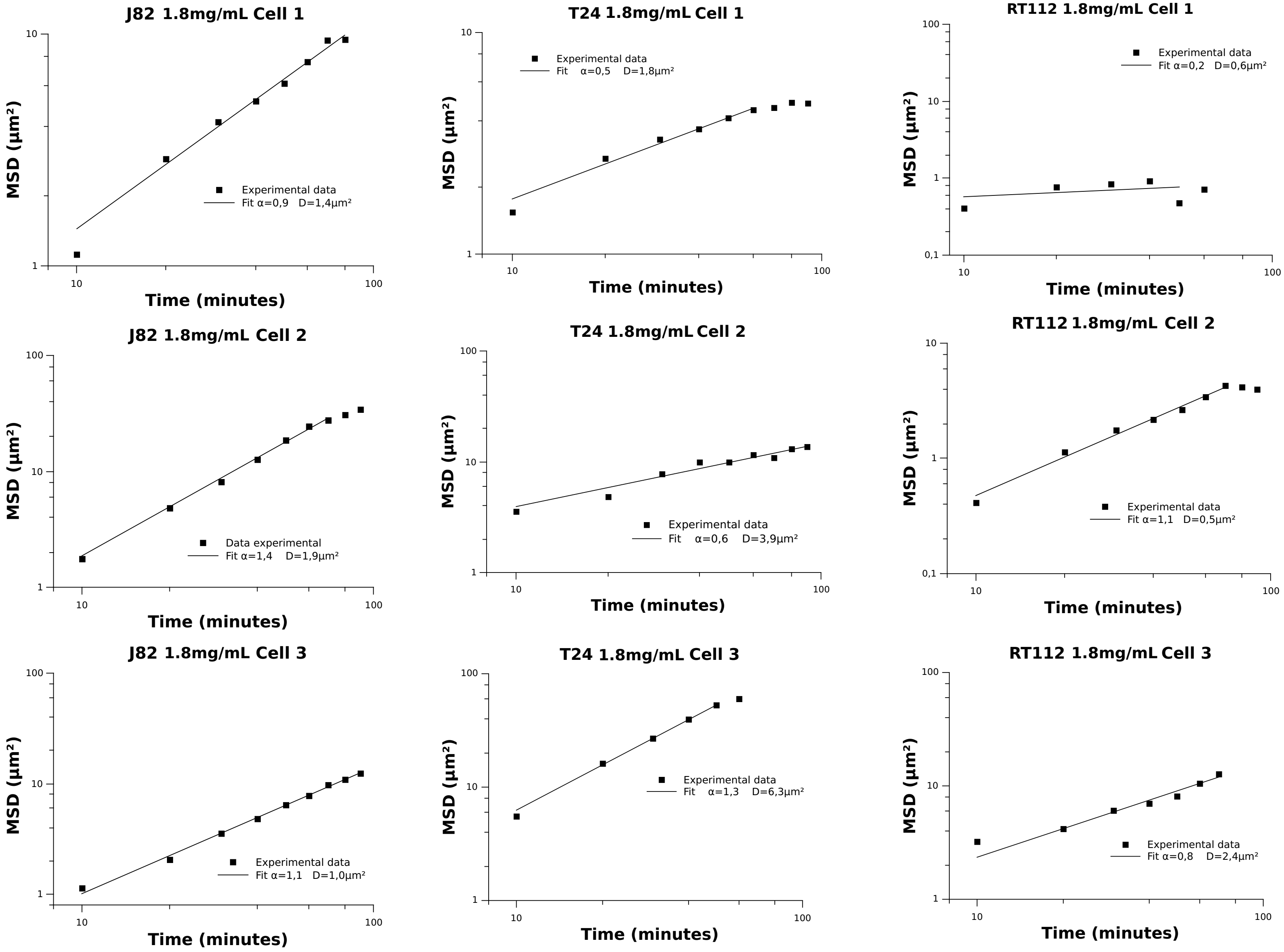

J82 1.8mg/mL Cell 1
Experimental data
Fit α=0,9 D=1,4µm²
T24 1.8mg/mL Cell 1
Experimental data
Fit α=0,5 D=1,8µm²
RT112 1.8mg/mL Cell 1
Experimental data
Fit α=0,2 D=0,6µm²
J82 1.8mg/mL Cell 2
Data experimental
Fit α=1,4 D=1,9µm²
T24 1.8mg/mL Cell 2
Experimental data
Fit α=0,6 D=3,9µm²
RT112 1.8mg/mL Cell 2
Experimental data
Fit α=1,1 D=0,5µm²
J82 1.8mg/mL Cell 3
Experimental data
Fit α=1,1 D=1,0µm²
T24 1.8mg/mL Cell 3
Experimental data
Fit α=1,3 D=6,3µm²
RT112 1.8mg/mL Cell 3
Experimental data
Fit α=0,8 D=2,4µm²
MSD (µm²)
Time (minutes)

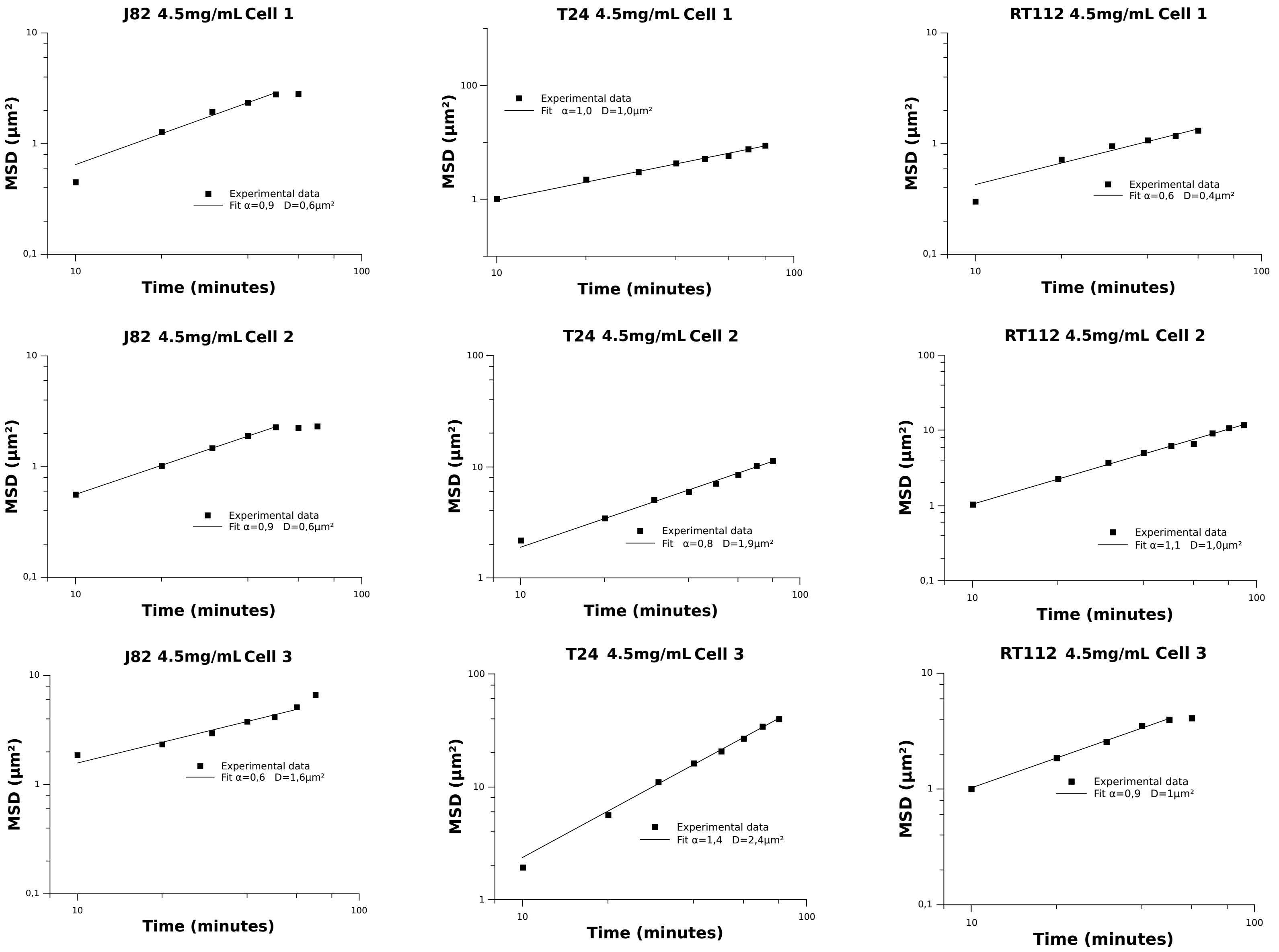
J82 4.5mg/mL Cell 1
Experimental data
Fit α=0,9 D=0,6μm²
T24 4.5mg/mL Cell 1
Experimental data
Fit α=1,0 D=1,0μm²
RT112 4.5mg/mL Cell 1
Experimental data
Fit α=0,6 D=0,4μm²
J82 4.5mg/mL Cell 2
Experimental data
Fit α=0,9 D=0,6μm²
T24 4.5mg/mL Cell 2
Experimental data
Fit α=0,8 D=1,9μm²
RT112 4.5mg/mL Cell 2
Experimental data
Fit α=1,1 D=1,0μm²
J82 4.5mg/mL Cell 3
Experimental data
Fit α=0,6 D=1,6μm²
T24 4.5mg/mL Cell 3
Experimental data
Fit α=1,4 D=2,4μm²
RT112 4.5mg/mL Cell 3
Experimental data
Fit α=0,9 D=1μm²
MSD (μm²)
Time (minutes)
