# Supplementary material for: Efficient deformation mechanisms enable invasive cancer cells to migrate faster in 3D collagen networks
Source: Sci Rep. 2022 May 12;12:7867. doi: 10.1038/s41598-022-11581-2 (PMC9098560; doi:10.1038/s41598-022-11581-2)
Supplement: Supplementary file 2 — Supplementary Legends. [file 41598_2022_11581_MOESM2_ESM.pdf]

Supplemental material: Movies S1 to S4 and Figure S5

- **Movie S1 – Confocal time sequence of T24 cell migration embedded in a 0.95 mg/mL collagen gel**. Superposition of collagen fibers is depicted in red. The 2D cell shape is shown in yellow. The time interval between 2 frames is 10 minutes.

- **Movie S2 – Time-lapse video of collagen fiber displacements during RT112 cell migration** The vector length and color indicate the displacement magnitude of collagen fibers in μm. The collagen concentration is 0.95 mg/mL and the time interval between two frames is 10 minutes. The initial 3D cell shape is shown in grey levels. The large arrow indicates the cell migration direction. The inset shows the initial F-actin location within the cell (projected Z-stack fluorescence of confocal images). The image number is indicated at the top left. The 3D orthonormal coordinate system is represented at the bottom left. The scale of the 3D grid surrounding the displacement field is displayed in μm.
  We observe a RT112 cell showing a slightly elongated morphology with no clear actin polarization. The migrating cell develops small extensions and its cytoplasm contains many actin aggregates (see fluorescent image inserts) similar to the amoeboid mode of migration. The largest fiber displacements are on the order of 2.6 μm (for a better visualization of fiber displacements, the maximum of the magnitude scale has been set to 2.1 μm). The cell migration causes scattered displacements of collagen fibers located behind or on the sides (see images #2, #3 and #5) : the cell removes these fibers. Fiber displacements located at the periphery of short extensions correspond to pulling by the cell (see image #1).

- **Movie S3 – Time-lapse video of collagen fiber displacements during T24 cell migration** The vector length and color indicate the displacement magnitude of collagen fibers in μm. The collagen concentration is 0.95 mg/mL and the time interval between two frames is 10 minutes. The initial 3D cell shape is shown in grey levels. The large arrow indicates the cell migration direction. The inset shows the initial F-actin location within the cell (projected Z-stack fluorescence of confocal images). The image number is indicated at the top left. The 3D orthonormal coordinate system is represented at the bottom left. The scale of the 3D grid surrounding the displacement field is displayed in μm.
  The T24 cell is highly polarized with a long cylindrical protrusion at the leading edge. The displacement magnitudes reach maxima around 3.6 µm. When the protrusion at the front extends, it pushes the collagen fibers (see image #1). As the back of the cell retracts, collagen fibers relax in this area (image #1). Then the cell undergoes a rest phase (image #2). But the cell also pulls on the collagen fibers at the front and on the lateral sides (image #3), in order to move forward (top of the figure). These local deformations are associated with the presence of dense actin regions (see fluorescent image insets). Next, the cell carries fibers located at the rear and lateral edges (images #4 and #5) to complete its motion.

- **Movie S4 – Time-lapse video of collagen fiber displacements during J82 cell migration** The vector length and color indicate the displacement magnitude of collagen fibers in μm. The collagen concentration is 0.95 mg/mL and the time interval between two frames is 10 minutes. The initial 3D cell shape is shown in grey levels. The large arrow indicates the cell migration direction. The inset shows the initial F-actin location within the cell (projected Z-stack fluorescence of confocal images). The image number is indicated at the top left. The 3D orthonormal coordinate system is represented at the bottom left. The scale of the 3D grid surrounding the displacement field is displayed in μm.
  The J82 cell has a very elongated shape with several large cylindrical protrusions. The main cylindrical protrusion displays several actin-rich regions on the lateral surface (see images #3 and #4). High concentrations of actin can also be seen at the end of protrusions and on the cell surface. The cell induces large displacement fields (maximum value of 7.2 µm) and the membrane is very dynamic. The cell pushes fibers at the front (images #1 and #4) and brings fibers to the back (images #1 and #2 and #5). We also observe the relaxation of fibers located near the end of protrusions (images #2 and #4).

- **Figure S5 – MSD Curves at different collagen concentrations for the three cell types**. The diffusion coefficient D and the exponent α are mentioned on each curve.
